# Reinventing chemotherapy

**DOI:** 10.1093/oncolo/oyae331

**Published:** 2025-02-27

**Authors:** Bruce A Chabner, Jacob Gehri, Beatrix B Thompson

**Affiliations:** Department of Medicine, Division of Hematology-Oncology, Massachusetts General Hospital, Boston, MA 02114, United States; Rothchild and Sons, Boston, MA 02114, United States; Harvard Medical School, Boston, MA 02114, United States

**Keywords:** chemotherapy, immunotherapy, antibody–drug conjugates, cancer pharmacology

At first glance, the future of chemotherapy as a standard component of cancer treatment is not promising. After 7 decades as the mainstay of adjuvant therapy and therapy for metastatic solid tumors, and providing cures for hematological malignancies, interest in infusional chemotherapy is clearly declining. In the past 5 years (2019-2023 inclusive), only one new cytotoxic agent, lurbinectedin, a DNA-binding natural product, has been approved in the US.^1^ In the same time period, many novel immunotherapies, including 4 checkpoint inhibitors, 8 bispecific antibodies, 8 antibody–drug conjugates (ADCs), and 4 new CAR-T regimens, as well as 30 novel targeted therapies have been approved. Thus, multiple new classes of cancer therapies have emerged as the dominant innovators in cancer treatment^2^ (Table 1). Since their initial approval in 2011, immune checkpoint inhibitors have received approval for more than 100 indications and have moved into first-line therapies for many solid tumors, both for metastatic disease and adjuvant therapy, and in many instances, replacing standard chemotherapy regimens. At the same time, CAR-T regimens are superseding high-dose chemotherapy for relapsed lymphoid malignancies. Among the lymphomas, first cured by chemotherapy regimens, anti-PD1 agents now have an established role in first-line therapy for Hodgkin lymphoma, improving survival and lessening the toxicity of curative regimens, and replacing bleomycin in multidrug chemotherapy regimens.^3^ For chronic lymphocytic leukemia, chronic myeloid leukemia, and many oncogene-driven solid tumors, targeted agents have replaced chemotherapy. The recent record of FDA approvals for cancer provides irrefutable evidence that cancer treatment is rapidly changing and that interest in immunotherapy and targeted therapy has largely replaced the prior focus on chemotherapy.

**Table 1.** Classes of new chemical entities approved as cancer therapeutics, 2019-2023.

Agent class	Number of new FDA approvals
Chemotherapeutic agent	1 (Lurbinectidin, 2019)
Antibody–drug conjugates	8
Bispecific antibodies	8
Checkpoint inhibitors	5
Targeted small molecules	30

The advantages of these new therapies, both immunotherapy and targeted therapy, are obvious: they are based on a rapidly expanding understanding of cancer biology, and therefore they carry the promise of greater tumor specificity, lesser toxicity, and, in the case of immunotherapy, evoking the potential of a powerful, and natural system of disease control. As with any new therapy, the management of toxicities, particularly for the checkpoint inhibitors, is challenging but constantly improving.^4^ Where does this leave our war horse, chemotherapy?

In fact, chemotherapy regimens, largely developed in prior decades, have adapted to the entry of immunotherapy and play a complementary role in combination with checkpoint inhibitors in the current treatment of lymphomas and common metastatic tumors, such as triple-negative breast cancer^5^ and non-oncogene addicted lung cancer.^6^ The combination of chemotherapy and checkpoint inhibition is now standard first-line therapy in multiple settings including in adjuvant/neoadjuvant therapy of these same tumors.^7,8^ The success of these combinations proves an important point, that chemotherapy, previously regarded as potently immunosuppressive, could partner effectively with T-cell-based therapies. Recent studies report early positive results for combinations of chemotherapy even with targeted therapies for EGFR-mutated lung cancer,^9^ and conclusive benefit for *FLT3*-positive acute myeloid leukemia.^10^ However, considering the dearth of new agents, traditional chemotherapy is ripe for replacement, and the primary hope for finding a role for drugs of this type may lie in the clinical development of chemotherapy as payloads for ADCs. While most recent ADC approvals have been for second or for third-line use, enfortumab, a nectin4-targeted antibody coupled with a maytansinoid payload, has been granted approval for first-line urothelial cancers, in combination with a checkpoint inhibitor, pembrolizumab.^11^

**Figure 1.**
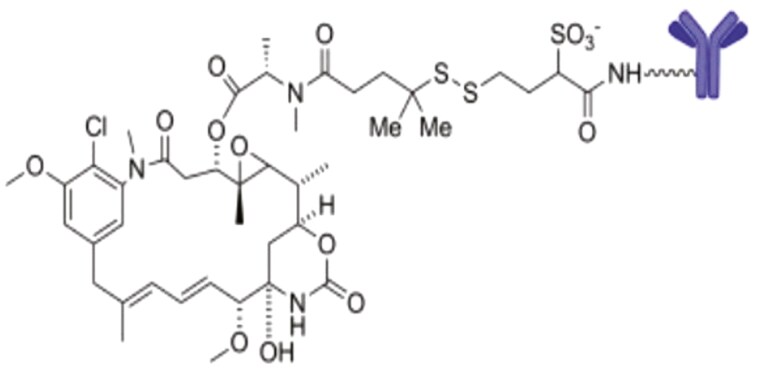
Structure of mirvetuximab soravtansine-gynx, an ADC composed of an antibody to the folate receptor α to which is linked the antimitotic maytansine^14^.

Serendipitously, the senior author (BAC) has had a personal if not peripheral role in the ADC story. In 1978 at the National Cancer Institute, BAC led the first-in-human trial of maytansine, a highly promising new inhibitor of microtubule synthesis.^12^ The trial was a failure because of the drug’s extreme gastrointestinal and neurological toxicity. In a classic oncology twist, what followed in the clinic was a lesser priority, paclitaxel, which proved difficult to develop but effective for multiple tumors. Several years after the maytansine trial, Ellwood et al., in a totally unrelated project in BAC’s laboratory, cloned the folate receptor,^13^ which is a folate and antifolate transporter. Other transporters proved more important for methotrexate and for pemetrexed, and the folate receptor was regarded as of little biological interest. However, it happened that the folate receptor is expressed on embryonic tissues, including the chorion, and on ovarian cancers and other tumors. and has resurfaced as the target of a newly approved ADC. A maytansinoid payload has been coupled to an anti-folate receptor antibody to make mirvetuximab soravtansine-gynx^14^ (Figure 1), an effective second-line drug for ovarian cancer.

Because of their targeted nature, ADCs have much greater activity as single agents as compared to the corresponding infusional chemotherapy agent. However, it is unlikely that single-agent ADCs will prove curative, as resistance emerges rapidly. Mechanisms of resistance are not well understood but include loss of membrane antigen expression, drug resistance related to the payload, and likely apoptotic alterations. To enhance their ability to cure, ADCs, following the lead of checkpoint inhibitors, are moving into adjuvant or neoadjuvant regimens for solid tumors, where the impact on tumor recurrence could be remarkable.^15^ An additional strategy will be the construction of multiagent regimens, including combinations of ADCs^16^ and ADCs with checkpoint inhibitors.^11^ The enfortumab–pembrolizumab experience, with an impressive 20% improvement in response rate in urothelial cancer as compared to the ADC alone, again teaches us that the co-administration of the ADC payload does not suppress the activity of T-cell mediated cell killing, which might have been an initial concern: the local release of chemotherapy (the so-called bystander effect) does not appear to suppress a T-cell response. The success of enfortumab with pembrolizumab raises the very attractive possibility of combining an ADC with other immune agents such as a bispecific antibody either using the drugs as separate molecules or even creating a single molecule featuring an ADC with a checkpoint inhibitor component. AZD 8205 is such an agent. Its antibody engages the checkpoint, B7–H4, which is highly expressed in many human epithelial cancers. AZD 8205 carries a topoisomerase I (top 1) payload and shows strong experimental activity against triple-negative breast cancer lines and xenografts.^17^

ADCs are not without toxicity, largely but not solely due to systemic release of the payload as the ADC circulates in the bloodstream or through reentry of drug from tumor cells into the circulation. Pharmacokinetic studies have documented the presence of cytotoxic levels (supra-nanomolar concentrations) of free SN-38 in patients receiving sacituzumab^18^ and free circulating deruxtecan in those treated with fam-trastuzumab deruxtecan.^19^ These studies suggest that the slow release of payload from bloodstream ADCs, which have a long plasma half-life, produces a lengthy payload exposure, leading to systemic toxicity, and potentially contributing to their antitumor action. In addition to their tumor-targeting properties, ADCs may have a multi-week half-life in the circulation, and thus a source of continuous drug exposure, a sort of constant infusion of free drug. The extent to which this prolonged drug exposure contributes to some of the unique toxicities and the antitumor activity is not known. Most prominent ADC toxicities are consistent with the known side effects of the payload class (antimitotics or topoisomerase I inhibitors). Others are unique and unanticipated. The basis for the unusual lung toxicity of fam-trastuzumab deruxtecan is not obvious; pneumonitis is not a prominent toxicity of other systemic top 1 inhibitors but was reported again in the phase I study of dapotamab, an ADC in which deruxtecan is linked to another antibody, an anti-trop2, suggesting that this particular toxicity may be intrinsic to the drug.^20^ A second possibility is that trastuzumab contributes to the lung toxicity in that pneumonitis has been reported with other trastuzumab ADCs and with trastuzuzmab alone.^21^ Other unexpected toxicities have emerged during early trials of ADCs, including the ocular toxicity of mirvituximab^14^ and the persistent, non-resolvable dermal, ocular, and pulmonary toxicity of pyrrolobenzodiazapine dimer in its initial trial as an HER-2 conjugate,^22^ leading to the discontinuation of trials of that ADC (DHES0815A).

There is significant interest in improving current ADCs, particularly in finding antibodies aimed at new tumor-specific targets. The claudins, membrane-associated ion channel proteins in embryonic cells, may be the next target for antibodies that gain drug approval, as a naked Claudin-18 antibody has shown significant activity in colon cancer trials^23^ and other claudins have become a prominent new target for ADC therapies in trial. Thus far, the number of targets of approved ADCs is relatively small; they are primarily B-cell lymphocyte related, Her2, TROP2, and nectin4, and the folate receptor but antibodies targeting many other tumor-associated antigens are under development.^21^

**Figure 2.**
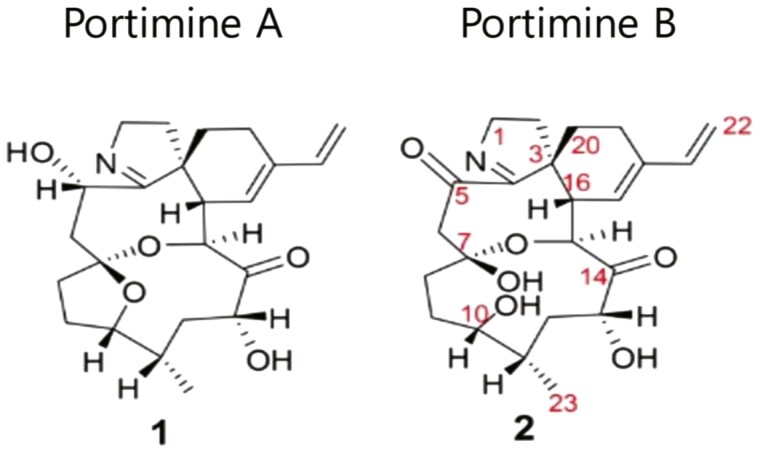
Structures of portimines A and B. The portimines were derived from mussels, exhibit picomolar antitumor activity in vitro, and a favorable therapeutic ratio in murine models^27^.

**Figure 3.**
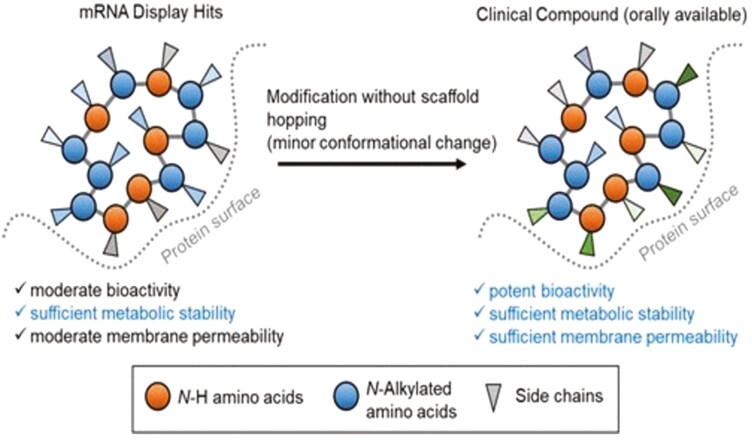
11-Mer cyclic peptide structures, containing extensively modified amino acids, orally bioavailable, and exhibiting picomolar inhibition of protein-protein and protein–DNA interactions^30^.

While most newly approved ADCs employ an antimitotic or topoisomerase 1 inhibitor, new payloads are being explored in clinical trials. Different, nonoverlapping toxicities will allow multi-agent ADC treatment with tolerable toxicity. While the majority of currently approved ADCs employ antimitotics (auristatin or maytansine analogs) or topoisomerase 1 (top1) inhibitors, there are many new candidate payloads with novel structures and promising features.^24–26^ Prominent among the newer payloads are compounds of marine origin.^25^ The auristatins are derived from dolastatin 10, an antimitotic isolated from a shell-free molusik. Vendotin (MMAE) is one of a family of monomethyl auristatin analogs, more than 40 of which are in preclinical and clinical ADC development.^26^ These and other marine products attract particular interest because of their picomolar antitumor potency, their stability in the acid environment of the lysosome, and their favorable linking properties. The hunt for new payloads with unusual mechanisms of action and acceptable toxicity patterns continues to identify candidate marine toxins, particularly those with novel mechanisms of action such as aplidin, an inhibitor of protein elongation factor eEF1A2,^26^ and the portimines, which as picomolar tumor cytotoxins, which have an apparent favorable therapeutic ratio in murine studies (Figure 2).^27^ Payloads other than traditional cytotoxic chemotherapeutics, including protein degraders,^28^ offer unique mechanisms of action and potentially nonoverlapping toxicities. Drug carriers with high affinity for tumor targets, such as nanobodies (derived from camel heavy chain peptides)^29^ and orally available cyclic peptides (Figure 3),^30^ will compete with antibodies as payload carriers for a place in cancer therapy.

As a final thought, if the goal of treatment is a cure, it would be a great advantage to have a *quantitative assessment* of treatment impact in the adjuvant and neoadjuvant settings, where the chances for cure are greatest. Currently, quantitation of tumor kill primarily depends on tumor measurement by imaging, circulating biomarkers, pathological assessment or in the case of hematological malignancies, molecular assessment of minimal residual disease (MRD). Treatment for solid tumors, with the exception of testicular cancers, is not guided by a highly sensitive assessment of MRD.^31^ While imaging provides the most important information for assessing therapy in patients with metastatic tumor, it is inadequate to detect residual subclinical disease. Advanced technologies such as ctDNA^31^ or circulating tumor cells^32^ are informative but have a substantial false negative rate, failing to detect significant numbers of patients with earlier stages of breast cancer or colon cancer who later develop recurrence.^33^ A significant improvement in sensitivity of ctDNA is needed. A recent report offers hope: while ctDNA has a short half-life of 1-2 h in plasma, in murine experiments, nanoparticles or DNA binding antibodies administered several hours prior to blood draws inhibited the degradation of ctDNA and elevated their levels in plasma 10-fold or greater.^34^ Particularly in the neoadjuvant and adjuvant settings, refinement of ctDNA assays,^35^ or alternative technologies such as detection of tumor-derived RNA in peripheral blood exosomes^36^ would enhance our ability to identify and limit treatment to patients at a high risk of recurrence, to judge the impact of treatment on minimal residual disease and would aid in picking the most effective agent and regimen. Improvements in sensitivity of residual tumor detaction would dramatically change many aspects of solid tumor therapy.

In conclusion, chemotherapy will survive and even flourish, but in a different context, as a component of an ADC. It is only a matter of time that these new entities will move into first-line therapy and replace much of current traditional infusional chemotherapy.
